# Spatial ecology to strengthen invasive snake management on islands

**DOI:** 10.1038/s41598-023-32483-x

**Published:** 2023-04-25

**Authors:** Borja Maestresalas, Julien C. Piquet, Marta López-Darias

**Affiliations:** Island Ecology and Evolution Research Group, Instituto de Productos Naturales y Agrobiología (IPNA-CSIC), 38206 La Laguna, Tenerife, Canary Islands Spain

**Keywords:** Herpetology, Conservation biology, Invasive species

## Abstract

Knowledge on the spatial ecology of invasive predators positively contributes to optimizing their management, especially when involving cryptic and secretive species, such as snakes. However, this information is lacking for most invasive snakes, particularly on islands, where they are known to cause severe ecological and socio-economic impacts. This research is focused on assessing the spatial ecology of the California kingsnake (*Lampropeltis californiae*) on Gran Canaria to strengthen management actions. We monitored 15 radio-tagged individuals once per day on 9–11 days per month from July 2020 to June 2021 to calculate the species' home range and describe annual activity patterns in the invaded range. To account for the species' diel activity during the emergence period, we additionally monitored snakes from January to May 2021 during three consecutive days per month in four different time intervals each day. We detected movement (consecutive detections at least 6 m apart) in 31.68% of the 1146 detections during the whole monitoring period. Movements most frequently detected were shorter than 100 m (82.24%), and among them the range 0–20 m was the most recurrent (27.03%). The mean distance of movement was 62.57 ± 62.62 m in 1–2 days. Average home range was 4.27 ± 5.35 ha—calculated with the Autocorrelated Kernel Density Estimator (AKDE) at 95%—and did not significantly vary with SVL nor sex. We detected an extremely low value of motion variance (0.76 ± 2.62 σ^2^m) compared to other studies, with a general inactivity period from November to February, January being the less active month of the year. Diel activity was higher during central and evening hours than during early morning and night. Our results should be useful to improve control programs for this invasive snake (e.g., trap placement and visual survey guidance) on Gran Canaria. Our research highlights the importance of gathering spatial information on invasive snakes to enhance control actions, which can contribute to the management of secretive invasive snakes worldwide.

## Introduction

Biological invasions have a broad range of impacts^1^ that are particularly harsh on islands^2,3^. Oceanic islands, priority areas for global biodiversity conservation^4^, host a great proportion of endemism^5,6^, while being extremely vulnerable to invasive species, especially predators^2,5^. Therefore, predator control or eradication on islands can provide numerous ecological benefits to insular ecosystems (e.g., Jones et al.^7^). However, invasive species management remains a complex task^8^, as its success and optimization depends on the available data about target species.

Information on the biology and ecology of invasive predators can be crucial to develop control strategies and action plans^8,9^, but particularly home range, activity patterns, or habitat use^10,11^ can facilitate more effective trapping^12,13^. This strategic information becomes even more relevant to organisms difficult to manage, like snakes^14,15^, which show extremely low detectability, secretive behavior, cryptic coloration, sporadic activity patterns, or use inaccessible habitats^16–18^. As invasive snakes are becoming increasingly recognized as a major threat to biodiversity on numerous islands worldwide^19,20^, understanding their spatial ecology is key to optimizing control actions^10,21,22^. However, such important details remain unknown or poorly studied for most of the world’s invasive snakes.

The present research focuses on the California kingsnake (*Lampropeltis californiae*), a medium-sized colubrid native to western United States and northern Mexico^23^, which was detected in 1998 on Gran Canaria (Canary Islands, Spain)^24^. Since then, the Canary Islands' and Gran Canaria governments have led yearly control actions, based on visual surveys and trapping from March to August each year^24,25^, yet the species is still expanding its invaded area^25^. Biological information on this species is scarce and mostly comes from its native range, where it is a diurnal, generalist and wide-foraging predator that feeds on terrestrial vertebrates—mainly snakes, lizards, mammals and birds^26^. In California, *L. californiae* has a relatively small home range (5.45 ± 5.97 ha) and a sporadic and marked seasonal activity, with a peak in spring^27,28^. This snake generally emerge from brumation in mid-February to early March^27,28^. Individuals remain underground most of the time^27^ in rodent burrows, under rocks, human structures or logs^28^. Activity generally occurs during the day, although it depends on daytime temperatures^28^. During warm days, they may only be active at night, while milder days allow morning or late afternoon activity^28^. On Gran Canaria, *L. californiae* usually preys on the three endemic reptiles of the island, along with rodents and followed by birds^29,30^, causing a severe impact on the herpetofauna and ecosystems^31,32^. A similar phenology to that of the native range can be inferred from the number of captures in the invaded range, which increases from March to May^24,25^. However, a scientific understanding of the spatial ecology of *L. californiae* in Gran Canaria is lacking but needed, since invasive species are known to shift their ecology or behavior once established in new environments^33,34^, and this information is essential to guide control actions^8^.

In this context, our study aimed to provide basic spatial ecology information, estimated home range and described year-round phenology and diel activity of *L. californiae* on Gran Canaria. We also analyzed whether home range and year phenology depended on body size or sex, and explored differences in diel activity between sexes and months. These comparisons are not only interesting from an ecological point of view, but can also inform managers to improve the design of control actions. Notably, they could focus on increasing captures of sexes or sizes that are more infrequently collected (i.e., females and small or large-sized individuals^24,25^). Exploring diel activity more in depth should also reveal the most appropriate hours to conduct visual surveys. Based on reports from the native range, we hypothesized that *L. californiae* should show overall small home ranges varying between sexes, males showing larger areas. We also anticipated a marked seasonal and diurnal activity—spring and the warmest hours of the day being the periods with higher activity—but with a shorter torpor period than in the native range, due to the mild weather of Gran Canaria all year round. Our research should be useful to guide managers in designing more efficient control actions to fight against this pernicious invader. From a global perspective, we expect our research to highlight the value of movement ecology to improve the management of secretive invasive predators.

## Materials and methods

### Experimental design: study area and sample size

Aiming to conduct a year-round monitoring of *L. californiae* spatial behavior, we located our study area in Amagro Mountain Natural Monument (NW Gran Canaria), invaded since 2010^24^, and where there were no anthropic infrastructures that would limit our monitoring or affect snake spatial behavior^35^.

In order to set our sample size, we had to adjust it to logistic and economic constraints, as well as to the number of snakes captured in Amagro and their weights. We only selected snakes captured in this area by the control staff between February and June 2020 to prevent homing behavioral effects^36^, and weighing over *c.* 180 g—transmitters (SI-2 9g, Holohil Systems, Ltd., Ontario, Canada) could only make up < 5% of animal weight^37^. We finally tagged 15 adult *L. californiae* individuals, measured their snout-to-vent length (SVL) with a measuring tape, and sexed them using a probe^38^. They comprised 6 males and 9 non-gravid females (mean ± SD SVL: 96.62 ± 11.05 cm; min–max SVL: 80–121 cm), all showing normal coloration^28,39^. All individuals were kept in captivity in appropriate conditions until surgical insertion of transmitters (all cases between July 4th–6th 2020), with a food intake the previous week. Surgery was carried out by an experienced veterinarian, following a protocol adapted from Melián^40^ (see Supplementary Material 1). Veterinarian post-surgery care lasted for 48–72 h. All individuals survived surgery, so we released them back into the wild in their capture location or its vicinity.

### Field tracking

To estimate home range and describe the species phenology, we tracked individuals from July 2020 to June 2021. This one-year survey involved one field tracking session per month, each of 9–11 consecutive days, with *c*. 15 days between sessions, detecting all individuals once per day. Complementarily, to confirm activity during night-time, early morning or dusk, as suggested by Hubbs^28^, we analyzed diel activity following an experimental design adjusted from Abom et al.^41^. Thus, we detected individuals four times a day during three consecutive days (starting on the third day of tracking described above), only from January 2021 to May 2021—i.e., emergence period until the number of captures starts to decrease^25^. Each day we detected individuals at the beginning of the following periods: (1) early morning (7:30–10:30), (2) central hours (10:30–17:30), (3) evening (17:30–20:30), and (4) night (20:30–7:30) (see Abom et al.^41^). Period duration is approximate, as we based session times on sunrise and sunset time each month.

To locate each individual exactly, a team of 2–3 people, each provided with a Biotrack three-element Yagi antenna coupled to a Biotrack Sika handheld receiver (Biotrack, UK), followed the signal until the 2–3 antennas coincided at the same spot. We recorded detections with a 5G GPS cell phone at *c.* 2 m precision, using the area aerial photograph in the QField App (The QField Project/OPENGIS.ch 2019). For each detection, we recorded whether each individual was on the surface (basking or moving) or sheltered.

### Data analyses

#### Data preparation

To estimate home range and describe the species phenology, we discarded the July 2020 data to avoid the collateral effects of surgery on movement behavior^42^. We defined movement as consecutive detections at least 6 m apart. An individual was assumed dead when it was detected in the same place for a long time (*c.* 1 month, excluding the brumation period, *c.* from November to February) until the end of the monitoring, assigning the date of the first detection at that place as the final detection date for subsequent analyses. Particularly for the diel activity analysis, we considered that an animal was active not only when it was located at least 6 m apart from the previous detection, but also when it was on the surface (informative of the opportunity to capture snakes for control purposes).

#### Tracking summary and basic spatial information

We extracted information on days that we tracked individuals, battery life, number of detections (basking/moving *vs.* sheltered), the percentage of detections that reflected movement, types of movements (classified by distance categories following Anguiano and Diffendorfer^27^), mean and maximum distance covered in 1–2 days, maximum time without performing movements, and frequency of movements. We calculated all distances as Euclidean.

#### Home range calculation

We calculated home range values with Autocorrelated Kernel Density Estimators (AKDE)^43^, a computation aid that takes into account tracking data autocorrelation while robustly dealing with data gaps and irregular sampling schedules^44^. To fit movement models, we used the *ctmm* v.0.6.1 package^45^, with the perturbative hybrid REML method (pHREML), and AICc to select the best fitting model^46^. Using the function ‘akde’ in the *ctmm* package^45^, we estimated weighted AKDE home range areas at the 95% contour, due to heterogeneous periods between samplings^44^. To check for AKDE's assumptions on range residency^47^, we first built up individual variograms using the *ggplot2* v.3.35 package^48^ and visually decided which ones we considered were stable. Secondly, we explored the effective sample size of all individuals (DOF area resulting from the ‘akde’ function) and retained for home range calculations only those with a value greater than 10 (following Montano et al.^49^). We also calculated home range using the Minimum Convex Polygon (MCP)^50^ (100%, 95%, and 50%) and Kernel Density Estimators (KDE)^51^ (95% and 50%) to allow comparisons with previous studies. However, we did not perform further analyses with these estimates as both are currently considered inappropriate^44^ (see Supplementary Material 2 for MCP and KDE results).

#### Species phenology

We analyzed activity patterns throughout the year via the motion variance parameter (a measurement of animal movement intensity) calculated with dynamic Brownian Bridge Movement Models (dBBMMs)^52^ using the *move* package v.4.0.6^53^. We set window size and margin parameters to 7 and 3 telemetry detections (7 and 3 days in our case), respectively, based on the typical movements of individuals (snakes moved every *c.* 3 days) and our sampling regime, as suggested by Kranstauber et al.^52^. Analysis of motion variance informs about behavioral changes across a tracking period, detailing how straight a movement path is, as well as how much a path varies in speed, and the scale of movements in a time window^52^. Motion variance is high when animals are active and their path is irregular, but low when animals are inactive and/or follow a regular path^52^.

#### Effect of SVL, sex and month in the species spatial ecology

To analyze the influence of SVL and sex on home range and motion variance, we performed Spearman’s correlation tests and Kruskal–Wallis tests^54^, respectively. We first checked home range and motion variance normality following the methods of Zuur et al.^55^, and the homogeneity of the variance for males and females with Levene's tests^56^. For motion variance, we additionally quantified individual variation with a repeatability estimation test^57^ using the *rptR v.* 0.9.22 package^58^, and seasonal patterns using month as the explanatory variable in a Welch's heteroscedastic *F* Test with trimmed means (rate set at 0.1) and Winsorized variances^59^ performed on the *onewaytest* package^60^.

We assessed snakes’ diel activity with a Generalized Linear Mixed-Effects Model (GLMM) using a binomial error distribution for activity occurrence, including sex, month, and period of day as fixed factors, and the individual as a random factor. We also tested the interaction between month and period of day as it was our primary interest, but discarded it due to convergence issues. After verifying model assumptions—i.e., homogeneity of variance, normality and dispersion of residuals—with *DHARMa* v.0.4.3 package^61^, we retrieved the models' main effects using type-II Wald Chi square tests performed with ‘Anova’ function^62^, conducting post hoc analyses with the *emmeans* v.1.7.0 package^63^ to obtain differences between each factor level.

We performed all analyses using R v.4.1.1^64^, presenting all results as mean ± SD.

### Ethical statement

Each procedure complied with relevant laws and institutional guidelines. Experimental protocols were all approved by the University of La Laguna Ethical Committee (Authorization no. 2978440) and later authorized by the competent institution, the Government of the Canary Islands (Authorization no. 133/2020 and 159/2021). All experiments complied with the ARRIVE guidelines (https://arriveguidelines.org). Thirty minutes before surgery, each individual received morphine as anesthetic agent, and IM medetomidine and IV alphaxalone as an anesthesia inducing agent (see Supplementary Material 1). No animal was sacrificed during this study.

## Results

### Tracking summary of *Lampropeltis californiae* on Gran Canaria

We tracked individuals for a mean of 220.20 ± 96.56 days (Table 1). We lost 13% of transmitter signals in March 2021 and 33.33% in both April and May 2021, with only three transmitters remaining active in June 2021 (one of them inserted in an individual that was dead by that time). Average transmitter battery life was 302 ± 23.36 days (12 months, ranging 6–18 months, according to the manufacturer; Holohil Systems, Ltd., Ontario, Canada). Four individuals died during our monitoring (310, 550, 670 and 930), two of which (310 and 550) were excluded from all analyses as they stopped moving in August and September 2020, respectively (Table 1). We added individual 670 to all analyses as it died in March 2021 and accumulated enough data, while individual 930 was only included in motion variance analysis as it did not show enough effective sample size.

**Table 1:** Tracking summary by individual, showing the first day of tracking (Start monitoring date) and the last day we included in our analysis (Last fix date).

ID	Start monitoring date	Last fix date	Sample period	Battery life	No. detections (July 2020)	Total movements (July 2020)	Short moves (2 to 6 m)	Mean distance (m)	Maximum distance (m)	Max. days without movements
010	09/07/20	12/05/21	279	310	98 (10)	28 (2)	9	76.34 ± 99.65	438.09	91
149	08/07/20	09/05/21	276	308	98 (11)	24 (2)	7	64.62 ± 50.49	160.92	39
190	08/07/20	03/06/21	301	333	100 (11)	35 (1)	3	37.37 ± 25.91	97.94	86
230	08/07/20	15/04/21	252	284	92 (11)	28 (1)	3	72.60 ± 66.77	289.56	59
270	08/07/20	12/05/21	279	311	99 (11)	41 (1)	5	88.73 ± 96.49	382.58	62
310	08/07/20	09/09/20	34	311	13 (11)	4 (2)	1	27.16 ± 18.45	40.75	–
350	08/07/20	03/06/21	301	333	100 (11)	35 (2)	6	46.30 ± 33.47	112.90	58
510	08/07/20	28/04/21	265	297	93 (11)	43 (2)	5	94.21 ± 55.58	235.45	9
550	08/07/20	06/08/20	0	297	0 (11)	0 (3)	0	–	–	–
590	07/07/20	12/05/21	279	312	99 (12)	26 (1)	0	28.79 ± 20.74	67.32	107
670	07/07/20	17/03/21	223	285	81 (12)	21 (3)	6	40.24 ± 35.67	128.18	9
710	08/07/20	17/03/21	223	255	81 (11)	23 (1)	6	49.85 ± 32.66	122.66	121
790	07/07/20	25/03/21	231	264	68 (12)	18 (1)	4	52.70 ± 38.76	134.85	122
930	07/07/20	09/11/20	95	334	31 (12)	17 (1)	0	81.03 ± 89.01	309.26	3
950	07/07/20	28/04/21	265	298	93 (12)	20 (4)	12	45.43 ± 40.43	139.20	32
Mean ± SD			220.20 ± 96.56	302 ± 23.36	76.40 ± 33.69	24.20 ± 12.00	4.47 ± 3.46	62.57 ± 62.62	189.98 ± 121.34	61.38 ± 41.90

Since we excluded July from the analyses, sample period represents the number of days between August 2020 and the last fix date that we used for each individual analysis. Battery life was calculated as the difference between the date the transmitters were implanted (4, 5 or 6 of July 2020) and the last day we received signal or the last monitoring day. We also show data points, movements (no. of movements longer than 6 m), and short movements (no. of 2 to 6 m movements) for each animal (July data between parentheses). We indicate movement mean ± SD (standard deviation) and maximum distance (m) (Euclidean) for each animal. We show the longest period (days) without movements we could ensure for each individual (Max days without movement). We also indicate overall mean ± SD for each parameter, except the dates.

Monitoring resulted in a total of 1146 detections for all 15 individuals (76.40 ± 33.69 per individual; Table 1), 31.68% of which occurred after a movement (> 6 m) (Table 1). Movements most frequently detected were shorter than 100 m (82.24%), and among them the range 0–20 m was the most recurrent (27.03%) (Supplementary Material 3, Table S3.1). Mean distance covered in 1–2 days by individuals during the entire period was 62.57 ± 62.62 m, with the maximum distance covered by an individual in one day being 438.09 m (Table 1). Maximum time without moving (> 6 m) averaged 61.38 ± 41.90 consecutive days (min–max: 3–122 days), occurring between November and February for all individuals, although we detected short movements (2–6 m) all through the tracking period (Table 1). After brumation, movement usually occurred in blocks of consecutive days, averaging 2.57 ± 1.70 days (min–max: 1–6 days). Animals were sheltered in 95.99% of the detections.

### Home range

**Figure 1:**
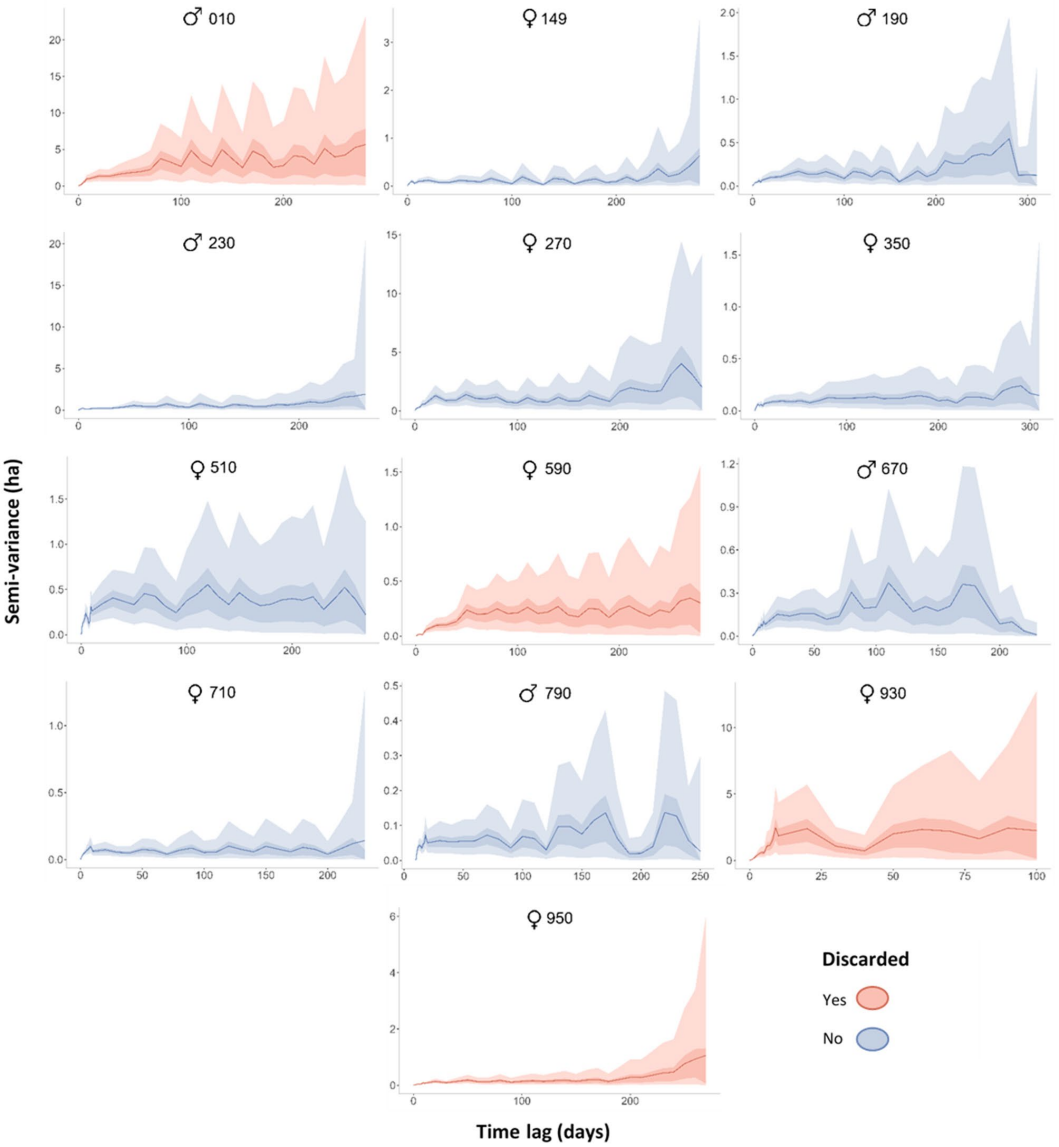
Variograms of the semi-variance at 50% (dark) and 95% (light) confidence intervals of each individual of *Lampropeltis californiae* in Gran Canaria. We show individuals remaining in the analyses in blue and those discarded in coral. We also indicate each individual's sex. We do not present information on dead 310 and 550, as we discarded them from all analyses.

**Figure 2:**
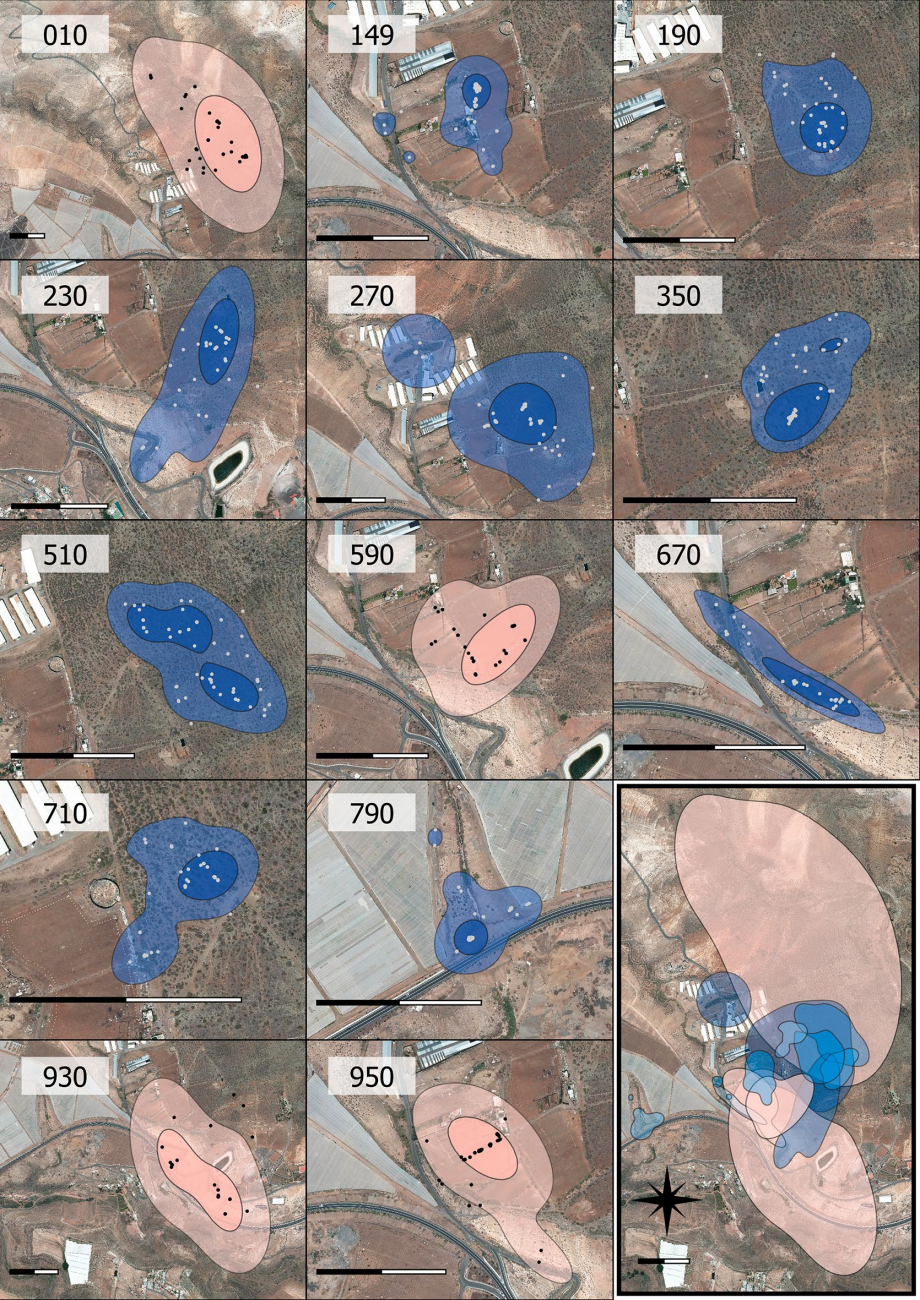
Estimates of the 50% (dark) and 95% (light) AKDE home range contours of each individual of *Lampropeltis californiae* in Gran Canaria. We show individuals remaining in the analyses in blue and those discarded in coral. Dots represent all detections of each animal. The whole scale bar represents 200 m in each representation. Last section shows 95% AKDE home range contours of all individuals, with discarded individuals in coral. We do not show information on dead 310 and 550, as we discarded them from all analyses.

We considered that all individuals’ variograms were stable (Fig. 1). However, the effective sample size of four individuals was exceptionally low (< 10; 010, 590, 930, 950) (Table 2), so we excluded them from home range analyses. Average *L. californiae* AKDE 95% contour home range was 4.27 ± 5.35 ha (males: 2.86 ± 2.52 ha, females: 5.40 ± 6.99 ha; Table 2, Fig. 2). There was no correlation between home range and SVL (*r*_*S*_ = -0.37, *P* = 0.323), nor significant difference between sexes ($\upchi_{1}^{2}$= 0.24, *P* = 0.624).

**Table 2:** AKDE (Autocorrelated Kernel Density Estimators) 95% home range areas (ha) per individual showing the lower and upper confidence intervals (CI) at 95%.

ID	Sex	SVL	Total detections	AKDE 95% home range	CI	Model	ESS	Discarded
010	Male	121	98	68.67	(19.70–147.63)	OUF anisotropic	4.26	Yes
149	Female	87	98	2.12	(1.49–2.85)	OU isotropic	37.18	No
190	Male	80	100	3.24	(1.77–5.14)	OU anisotropic	14.07	No
230	Male	89	92	6.28	(3.66–9.60)	OU anisotropic	16.98	No
270	Female	97	99	17.68	(11.47–25.21)	OU isotropic	25.29	No
350	Female	90	100	1.62	(1.09–2.25)	OU anisotropic	29.57	No
510	Female	90	93	4.52	(3.24–6.01)	OU anisotropic	40.69	No
590	Female	95	99	4.90	(1.59–10.06)	OUF anisotropic	4.97	Yes
670	Male	100	81	0.85	(0.46–1.37)	OU anisotropic	13.1	No
710	Female	90	81	1.03	(0.73–1.38)	OUf anisotropic	38.99	No
790	Male	107	68	1.08	(0.73–1.49)	OU isotropic	31.1	No
930	Female	110	31	29.95	(13.59–54.69)	OUf anisotropic	7.6	Yes
950	Female	100	93	3.86	(1.50–7.33)	OU anisotropic	6.58	Yes

We also indicate sex, snout-vent length (SVL) (cm), number of data points, effective sample size (ESS; parameter based on the number of range crossings occurred during the study period) and the best fitted movement model per individual. Movement models are OU: correlated positions but uncorrelated velocities, OUF: correlated positions and correlated velocities, isotropic: movement in all directions (circular home range) and anisotropic: movement vary by direction (non-circular home range)^103,104^. OUf is a particular case of OUF where autocorrelated positions and velocities cannot be distinguished^103,104^. Discarded individuals are noted. We do not show information on dead 310 and 550, as we discarded them from all analyses.

### Activity patterns

**Figure 3:**
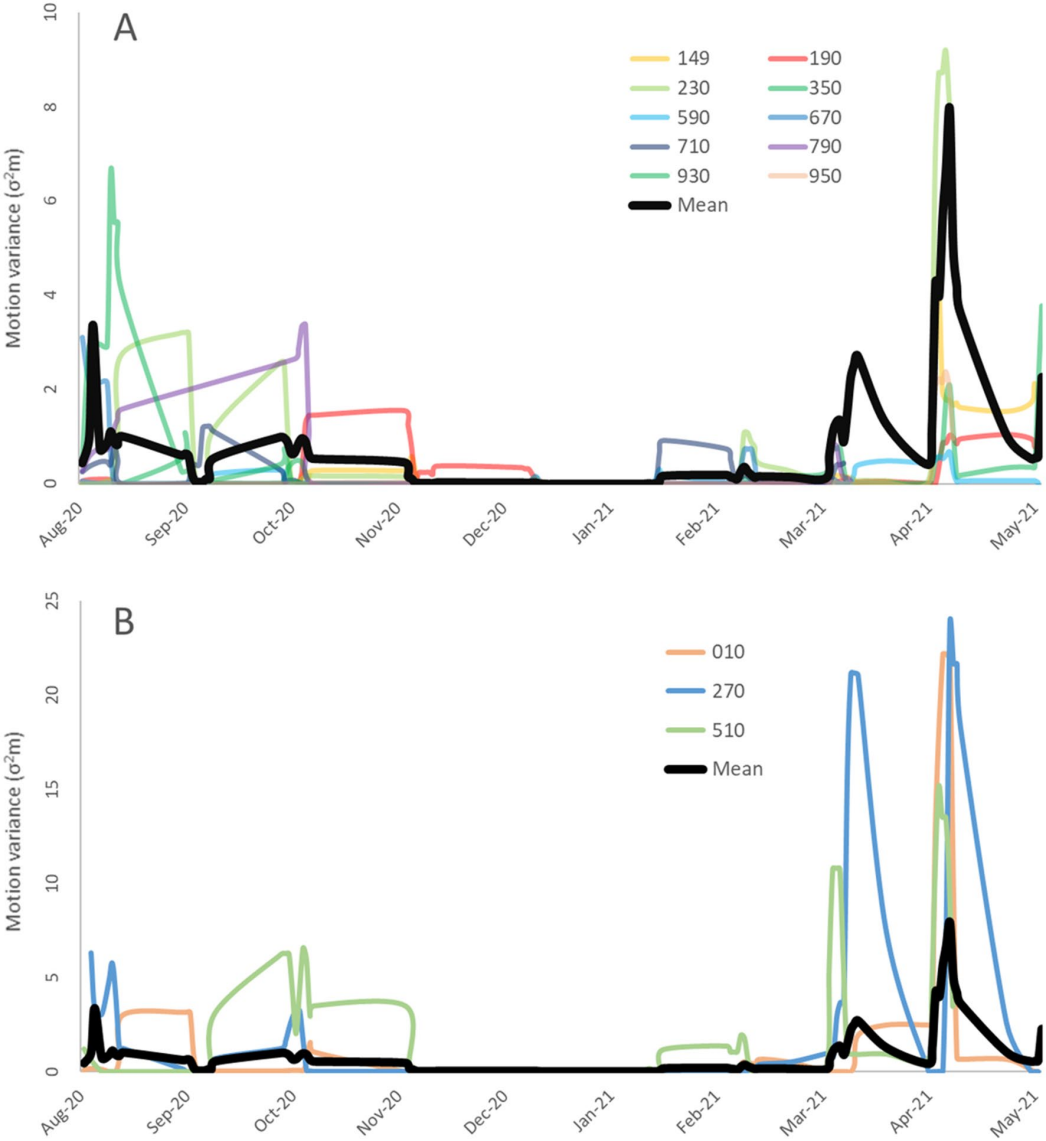
Motion variance values (σ^2^m) along the whole year tracking period colored by individuals. Black line represents the mean value of motion variance for all individuals. Individuals with a motion variance close to the mean are shown in **A**, whereas **B** shows those with higher values. Individuals discarded for this analysis (310 and 550) are not shown.

Average motion variance was 0.76 ± 2.62 σ^2^m during the whole period, although individuals exhibited consistent and significant differences (*R* = 0.09, *CI* = 0.03–0.17, *P* < 0.005). No significant correlation existed between motion variance and SVL (*r*_S_ = − 0.016, *P* = 0.608), nor differences between sexes (males: 0.62 ± 2.29 m, females: 0.85 ± 2.81 m; $\upchi_{1}^{2}$ = 1.25, *P* = 0.263). Motion variance significantly differed among months (*F*_9, 210.78_ = 8.45, *P* < 0.005; Fig. 3).

**Figure 4:**
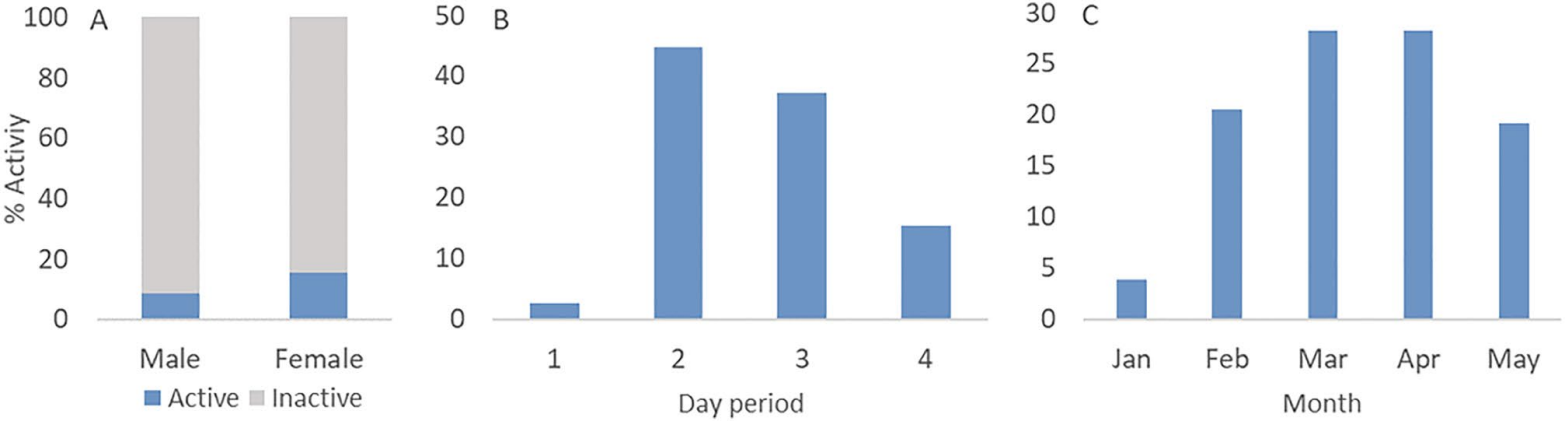
Diel activity of *Lampropeltis californiae* from January to May 2021. **A** represents the percentage of activity divided by sex. **B** and (**C**) represent the percentage of activity divided by period of the day and month, respectively.

Results from the GLMM revealed that diel activity was significantly affected by sex ($\upchi_{1}^{2}$ = 4.26, *P* = 0.039), month ($\upchi_{1}^{2}$ = 20.03, *P* < 0.005), and also period of day ($\upchi_{1}^{2}$ = 29.51, *P* < 0.005) during the intense monitoring (January-May 2021). Females were more frequently active (basking/moving) than males (15.68% female detections and 8.55% male detections) (Fig. 4A). Post-hoc comparisons between months showed that January was the month with a significantly lower activity (*P* < 0.05 for all comparisons: Fig. 4B). Snakes were significantly more active during the central hours of the day and the evening than in the early morning and night (*P* < 0.05 for all comparisons; Fig. 4C).

## Discussion

Our research provides essential information on the spatial ecology of the invasive *L. californiae* on Gran Canaria, which contributes to guide control actions for this pernicious invader. Home range data can be incorporated into designing control actions to manage invasive terrestrial vertebrates^10,12,65^, and are key to defining the density and distribution of traps^13^. On Gran Canaria, trap placement did not follow a specific density and distribution. Extracted from our home range calculation, to maximize the probability of a snake encountering a trap, these should not be separated by more than the minimum range detected, 104 m (diameter of a 0.85 ha circle, assuming a circle to be the most parsimonious design for a home range area; see Roy et al.^66^ for this calculation). Since this action is resource-consuming, trap separation could alternatively be no more than 233 m, based on the average home range detected (4.27 ± 5.35 ha). In relation to trap layout and based on previous studies^67,68^, we recommend using grids or linear designs, particularly in biodiversity-sensitive areas of Gran Canaria or along the expansion front. The use of drift-fences can also increase the probability of capture^69^. Distance traveled in a day can be used to infer the width of control buffers to prevent invasion of adjacent areas. Given that most movements detected were smaller than 100 m, we recommend a containment buffer width of 100 m. These recommendations can be improved in the future by calculating trap distance using simulations^65^ or the most cost-effective trap arrangement^70,71^. Control staff can also incorporate snake density information, as it could influence individual home range^12,35^.

*Lampropeltis californiae* phenology on Gran Canaria is highly seasonal, with a brumation period from late November to early February, which coincides with information reported for the island/coastal regions of its native range^28^. The species is active the rest of the year, with an activity peak between March and May, presumably linked to the breeding season^30^. Each year, environmental managers have reinforced control actions by hiring extra personnel between March and August. Following our results, this reinforcement should be extended from early February to mid-November, increasing efforts before the breeding season and until the end of the activity period. From an ecological perspective, the average motion variance of *L. californiae*, a wide-foraging predator^26^ (0.76 ± 2.62 σ^2^m, mean ± SD), is lower than in other active-searching or even ambush predators (mean ± SE for active predators: *Ophiophagus hannah:* 27.9 ± 0.6 m^72^, *Boiga cyanea:* 2.8 ± 0.8 σ^2^m^73^; mean ± SE for an ambush predator: *Python bivittatus*: 2.66 ± 0.14 m^74^). This seems to indicate that the species continuously forages until finding prey and then shelters and remains stationary for several days, lying dormant until it surfaces again to prey—which coincides with our findings that movements usually occurred in blocks of consecutive days. This result can be applied to management when a snake is detected but not captured, because the animal will probably remain in the same shelter/area for 2–3 days. Consequently, prospection of the surrounding area during that period could increase the probability of capture. In addition, as a notable amount of movements were short, intense visual surveys in the proximity of fresh snake tracks (e.g., scats or shed skins) for a similar amount of days can be also appropriate. Finally, it is worth noting that, since individuals were sheltered in most of the detections, the development of novel techniques to detect animals while immobile or sheltered is crucial to improve control success^67,75–77^. Due to visual surveys being extremely time- and resource-consuming^15^ for such a secretive snake, increasing detection on surface still requires further technological advances—e.g., remote sensing techniques^78^.

Diel activity analysis showed that females were more frequently active than males between January and May 2021, potentially due to their different behaviors during the breeding season (i.e., feeding, basking)^79^ or the associated reproductive costs of breeding for females^80^. This strengthens our previous recommendation to reinforce control staff from mid-February onwards to increase the probability of capturing females before reproduction begins. We also determined that *L. californiae* is mainly active during central hours and the evening, although a certain proportion of activity occurred at night during normal weather (15.40% of all activity detected), and even in the rain. To link visual surveys to species activity pattern^81^, the former should be made during central hours and evenings.

The overall spatial parameters studied for *L. californiae* on Gran Canaria showed a wide variation. This disparity may be explained mainly by the effect of individual heterogeneity on spatial behavior, already demonstrated for other invasive snake species^82^. This spatial heterogeneity can be due to individual body condition affecting movement ecology^83^, individual personality influencing exploratory and defensive behaviors^84^ or boldness and sociability^85^, as well as dispersive movements (such as may have happened with our individual 010). In addition, although some deaths are expected in this type of studies (e.g., due to tagging surgery, predators, health condition^see^ ^27,73,86^), we noted a higher death rate than expected. This reduced our final sample size, and possibly skewed the results obtained in some comparisons, mainly regarding sexes. Finally, several ecological parameters can also influence animal spatial behavior (for instance density, prey and shelter availability, degree of anthropization, habitat type^12,35,87^). We are however highly confident that our results provide robust knowledge on home range, phenology and activity patterns of *L. californiae,* which can be used to improve control action designs for the whole island of Gran Canaria and possibly elsewhere.

Technological progress is still needed to facilitate the acquisition of reliable spatial ecology data for small, low-mobile and secretive snakes. GPS-based techniques for the study of animal movement have greatly advanced in recent years^88^, including for invasive snakes^89^. Nevertheless, this technology is still difficult to apply to cases like ours that involve a fossorial, less mobile, and small-bodied species^89,90^. To accumulate enough effective sample size for this animal, small but long-duration batteries are needed, a trade-off that still remains defying (see Mitchell et al.^91^). Against this backdrop, the combined use of radiotelemetry and the novel AKDE home range estimation method allowed us to mitigate common telemetry data issues (e.g.*,* irregular sampling regimes, data autocorrelation) and those deriving from snake behavior (e.g., spatial autocorrelation, small effective sample size), without compromising data accuracy. Therefore, this combination is a promising tool to unveil the spatial ecology of other secretive species whose studies may encounter similar methodological difficulties arising from their particular behavior^92,93^.

From a broader perspective, our study contributes to highlight the advantages of gaining information on spatial behavior in the management of invasive species. Spatial ecology studies have enabled managers to understand invasive species' home range, activity patterns and habitat use^11,65^, can be also useful to plan when and where to place traps^94^ and conduct visual surveys^10^, determine sources of detection bias^15^, identify areas at risk^95^ or manage local habitats to prevent spread of invasive species^96^. Therefore, we argue that efforts should be made to turn spatial behavior information into an essential tool for optimizing invasive species management.

## Conclusions

The high ecological adaptability of *L. californiae*, its secretive behavior and unique propensity to survive^28^, its reproductive plasticity^97^, devastating ecological impacts^32,98^, and the climatic suitability of the archipelago^99^ makes the strengthening of actions to control this invasion an urgent matter for Gran Canaria. Our research provides basic and applicable insights into the spatial ecology of *L. californiae* that can be directly incorporated into trapping and control action designs. In particular, traps should not lie more than 233 m apart and containment buffers should be less than 100 m. Moreover, control action reinforcement should be extended from early February to mid-November to increase captures (particularly of females), that is, before the breeding season starts and until the end of the activity period. Since *L. californiae* individuals move every 2–3 days and most movements are smaller than 100 m, intense prospection in the surroundings of a detected but uncaptured individual or of fresh tracks could increase the probability of capture. To link visual surveys to the species activity patterns, the former should be made during central hours and evenings. In addition, this study underlines the value of spatial ecology in the context of biological invasions. Such an approach can be an essential step in designing more effective control strategies, especially on other islands worldwide (e.g., Cozumel, Christmas or the Balearic Islands^19,100–102^). Moreover, the combined use of AKDE method and radio tracking for less mobile small species like *L. californiae* is very appropriate. For a broader perspective, we appeal to the need to innovate and develop new technologies to improve management of invasive snakes, given their devastating impacts on many islands of the world.

## Supplementary Information

Supplementary Information.

## Data Availability

The datasets used and/or analyzed during this study are available from the corresponding author on reasonable request.
