# Supplementary material for: Spatial ecology to strengthen invasive snake management on islands
Source: Sci Rep. 2023 Apr 25;13:6731. doi: 10.1038/s41598-023-32483-x (PMC10130030; doi:10.1038/s41598-023-32483-x)
Supplement: Supplementary file 1 — Supplementary Information. [file 41598_2023_32483_MOESM1_ESM.docx]

spatial ecology to strengthen invasive snake management on islands

Borja Maestresalas^1^, Julien C. Piquet ^1^, Marta López-Darias^1*^

^1^ Island Ecology and Evolution Research Group, Instituto de Productos Naturales y Agrobiología (IPNA-CSIC), 38206 La Laguna, Tenerife, Canary Islands, Spain.

^*^ Correspondence author: [mdarias@ipna.csic.es](mailto:mdarias@ipna.csic.es); Tel.: +34 922474328

# **SUPPLEMENTARY INFORMATION 1**

Interventions started on July 4, 2020 and lasted 3 days. During the preoperative period, animals were kept at optimum temperatures of 24-30°C, with subcutaneous and tempered fluid therapy. The surgery table had a heating pad, and the operating room had all the sterility and aseptic conditions typical of this intervention. Thirty minutes before surgery, each individual was administered morphine as anesthetic agent, IM medetomidine and IV alphaxalone as an anesthesia inducing agent, and a broad-spectrum antibiotic (Cefotaxime). During the intervention, individuals remained intubated with inhalation of anesthesia (isofluorane with Ayres T circuit 2-3% isofluorane + 0.2-0.3 L / min O_2_ and forced ventilation). During the intervention, a celiotomy was performed on the last third of the animal that allowed the insertion of the transmitter, an incision that was subsequently closed by layers (skin, muscle and peritoneum) with U-shaped stitches, made with absorbable synthetic suture and closure with Vetbond (surgical glue). The postoperative protocol included the reversal of medetomidine to ensure autonomous respiration, the placement of the animal in an incubator at optimal body temperature, a recovery of 20-30 minutes, and a dose of analgesia and antibiotic at 48 and 72 hours, until the release.

SUPPLEMENTARY INFORMATION 2

**Table S2.1.** Home range area (ha) of each individual estimated with MCP (100%, 95% and 50%) and KDE *h_ref_* (95% and 50%)^1,2^, showing mean and SD of each estimator for all individuals. Information on individuals deceased in early stages of the monitoring (310 and 550) is not shown as these were discarded from all analyses.

| ID | Sex | SVL (cm) | MCP100 | MCP95 | MCP50 | KDE95 | KDE50 |
| --- | --- | --- | --- | --- | --- | --- | --- |
| 010 | Male | 121 | 15.76 | 15.59 | 3.84 | 34.75 | 7.08 |
| 149 | Female | 87 | 2.60 | 1.24 | 0.03 | 2.34 | 0.25 |
| 190 | Male | 80 | 1.95 | 1.22 | 0.20 | 2.31 | 0.34 |
| 230 | Male | 89 | 4.16 | 3.71 | 0.72 | 6.85 | 0.95 |
| 270 | Female | 97 | 20.33 | 13.20 | 0.05 | 16.40 | 2.08 |
| 350 | Female | 90 | 1.10 | 0.71 | 0.19 | 1.72 | 0.32 |
| 510 | Female | 90 | 3.06 | 2.71 | 1.41 | 5.58 | 1.52 |
| 590 | Female | 95 | 1.28 | 1.18 | 0.05 | 2.77 | 0.59 |
| 670 | Male | 100 | 0.35 | 0.30 | 0.02 | 1.82 | 0.42 |
| 710 | Female | 90 | 0.87 | 0.63 | 0.03 | 1.18 | 0.18 |
| 790 | Male | 107 | 1.21 | 0.35 | 0.00 | 1.13 | 0.12 |
| 930 | Female | 110 | 12.66 | 9.99 | 0.86 | 31.29 | 6.75 |
| 950 | Female | 100 | 1.90 | 0.35 | 0.00 | 1.30 | 0.16 |
| Mean |  |  | 5.17 | 3.94 | 0.57 | 8.42 | 1.60 |
| SD |  |  | 6.58 | 5.34 | 1.08 | 11.70 | 2.43 |

SUPPLEMENTARY INFORMATION 3

**Table S3.1.** Number and frequency of movement traveled for males and females *Lampropeltis californiae* in Gran Canaria, performed in 1-2 days, grouped by distance following the same categories as in Anguiano & Diffendorfer^3^.

| Movement  categories | Males | | Females | | Total | |
| --- | --- | --- | --- | --- | --- | --- |
|  | N | % | N | % | N | % |
| 6-20 | 26 | 29.55 | 44 | 25.73 | 70 | 27.03 |
| 21-40 | 19 | 21.59 | 29 | 16.96 | 48 | 18.53 |
| 41-60 | 10 | 11.36 | 26 | 15.20 | 36 | 13.90 |
| 61-80 | 12 | 13.64 | 16 | 9.36 | 28 | 10.81 |
| 81-100 | 10 | 11.36 | 21 | 12.28 | 31 | 11.97 |
| 101-120 | 5 | 5.68 | 10 | 5.85 | 15 | 5.79 |
| 121-140 | 2 | 2.27 | 8 | 4.68 | 10 | 3.86 |
| 141-160 | 1 | 1.14 | 5 | 2.92 | 6 | 2.32 |
| 160-180 | 1 | 1.14 | 4 | 2.34 | 5 | 1.93 |
| 181-200 | 0 | 0.00 | 3 | 1.75 | 3 | 1.16 |
| 201-220 | 0 | 0.00 | 0 | 0.00 | 0 | 0.00 |
| 221-240 | 0 | 0.00 | 1 | 0.58 | 1 | 0.39 |
| 241-260 | 0 | 0.00 | 1 | 0.58 | 1 | 0.39 |
| 261-280 | 0 | 0.00 | 0 | 0.00 | 0 | 0.00 |
| 281-300 | 1 | 1.14 | 0 | 0.00 | 1 | 0.39 |
| >300 | 1 | 1.14 | 3 | 1.75 | 4 | 1.54 |
| Total | 88 | 100.00 | 171 | 100.00 | 259 | 100.00 |

**References**

1. Mohr, C. O. Table of equivalent populations of North American small mammals. *Am. Midl. Nat.* **37**, 223–249 (1947).

2. Worton, B. J. Kernel methods for estimating the utilization distribution in home‐range studies. *Ecology* **70**, 164–168 (1989).

3. Anguiano, M. P. & Diffendorfer, J. E. Effects of Fragmentation on the Spatial Ecology of the California Kingsnake (*Lampropeltis californiae*). *J. Herpetol.* **49**, 420–427 (2015).
